# Chronic Chagas Cardiomyopathy Patients and Resynchronization Therapy: a Survival Analysis

**DOI:** 10.21470/1678-9741-2017-0134

**Published:** 2018

**Authors:** Antônio da Silva Menezes Junior, Cynthia Caetano Lopes, Patrícia Freire Cavalcante, Edésio Martins

**Affiliations:** 1 Escola de Ciências Médicas, Farmacêuticas e Biomédicas of the Pontifícia Universidade Católica de Goiás (PUC-GO), Goiânia, GO, Brazil.

**Keywords:** Chagas Disease, Cardiac Resynchronization Therapy, Heart Failure

## Abstract

**Introduction:**

Chagas disease represents an important health problem with socioeconomic impacts in many Latin-American countries. It is estimated that 20% to 30% of the people infected by *Trypanosoma cruzi* will develop chronic Chagas cardiomyopathy (CCC), which is generally accompanied by heart failure (HF). Cardiac resynchronization therapy (CRT) may be indicated for patients with HF and electromechanical dysfunctions.

**Objective:**

The primary endpoint of this study was to analyze the response to CRT in patients with CCC, while the secondary endpoint was to estimate the survival rates of CRT responder patients.

**Methods:**

This is an observational, cross-sectional and retrospective study. The records of 50 patients with CRT pacing devices implanted between June 2009 and March 2017 were analyzed. For statistical analyses, Pearson's correlation was used along with Student's t-test, and survival was analyzed using the Kaplan-Meier method. A *P* value of <0.05 was considered significant.

**Results:**

Out of 50 patients, 56% were male, with a mean age of 63.4±13.3 years and an average CRT duration of 61.2±21.7 months. The mean QRS duration was 150.12±12.4 ms before and 116.04±2.2 ms after the therapy (*P*<0.001). The mean left ventricular ejection fractions (LVEF) were 29±7% and 39.1±12.2% before and after CRT, respectively (*P*<0.001). A total of 35 (70%) patients had a reduction of at least one New York Heart Association (NYHA) functional class after six months of therapy (*P*=0.014). The survival rate after 72 months was 45%.

**Conclusion:**

This study showed clinical improvement and a nonsignificant survival rate in patients with CCC after the use of CRT.

**Table:** 

Abbreviations, acronyms & symbols				
ACE	= Angiotensin-converting enzyme		LVESV	= Left ventricular end systolic volume
ARB	= Angiotensin-receptor blockers		MADIT	= Multicenter Automatic Defibrillator ImplantationTrial
CARE-HF	= Cardiac Resynchronization-Heart Failure Trial		MIRACLE	= Multicenter InSync Randomized Clinical Evaluation
CCC	= Chronic Chagas cardiomyopathy		NHC	= National Health Council
CI	= Confidence interval		NYHA	= New York Heart Association
COMPANION	= Comparison of Medical Therapy, Pacing and Defibrillation in Heart Failure		PSVT	= Paroxysmal supraventricular tachycardia
CRT	= Cardiac resynchronization therapy		RAFT	= Resynchronization/Defibrillation for Ambulator Heart Failure Trial
HF	= Heart failure		RBBB	= Right bundle branch block
HR	= Hazard ratio		REVERSE	= The Resynchronization Reverse Remodeling in Systolic Left Ventricular Dysfunction
ICD	= Implantable cardioverter defibrillator		VT	= Ventricular tachycardia
LAFB	= Left anterior fascicular block		WHO	= World Health Organization
LBBB	= Left bundle branch block			
LVEF	= Left ventricular ejection fraction			

## INTRODUCTION

According to the World Health Organization (WHO), Chagas disease is an important health problem with socioeconomic impacts in many Latin-American countries^[1]^p>. Chagas disease, also known as American Trypanosomiasis, is caused by the protozoan *Trypanosoma cruzi*, and WHO estimates that there are approximately 8 million people infected worldwide^[1]^p>.

It is known that only 20% to 30% of those infected with *Trypanosoma cruzi* will develop chronic Chagas cardiomyopathy (CCC), one of its most frequent and severe clinical forms^[2,3]^p>. This disease causes loss of myocardial contractile function, dilation of cardiac chambers, and destruction of cardiomyocytes as well as fibrosis and scars. These patients may develop arrhythmias, apical aneurysms, thromboembolisms, sudden death, and heart failure (HF)^[3]^p>. The progression of HF often leads to changes in intracardiac electrical conduction, progressing with atrioventricular, intraventricular or interventricular conduction disorders^[4]^p>.

The pharmacotherapy agents used in the treatment of HF are well established and widely used^[5]^p>. Recently, in addition to pharmacotherapy, cardiac pacing has also been a treatment option with major impact on quality of life and reduction of mortality^[6]^p>.

In the last decade, several multicenter studies - such as COMPANION (Comparison of Medical Therapy, Pacing and Defibrillation in Heart Failure)^[7]^p>, CARE-HF (Cardiac Resynchronization-Heart Failure Trial)^[8]^p>, REVERSE (The Resynchronization Reverse Remodeling in Systolic Left Ventricular Dysfunction)^[9]^p>, MIRACLE (Multicenter InSync Randomized Clinical Evaluation)^[10]^p>, RAFT (Resynchronization/Defibrillation for Ambulatory Heart Failure Trial)^[11]^p> and MADIT (Multicenter Automatic Defibrillator Implantation Trial)^[12]^p> - were carried out in order to elucidate the indications and guidelines for cardiac resynchronization therapy (CRT) in patients with chronic HF^[13-15]^p>. However, these studies are not specific for CCC, as this etiology is not common in the countries in which these trials were conducted.

For the use of CRT, it is necessary to identify the patients who will respond to this therapy and those who will not. Studies show that responders and nonresponders can be identified by clinical (New York Heart Association [NYHA] functional class, 6-minute walk test, peak oxygen consumption, and quality of life score) and echocardiographic (improvement in left ventricular ejection fraction [LVEF], reduction in end-diastolic and end-systolic volume, and decreasing mitral insufficiency) parameters, as the measurements of these parameters have already been used to identify responders. However, there is no consensus in the literature about the relevant responses to this therapy^[16-22]^p>.

Thus, this study aimed to analyze the long-term responses of patients with CCC using CRT and to estimate the survival rate of responder patients.

## METHODS

This is an observational, cross-sectional and retrospective study. The medical records of patients with CCC who had cardiac resynchronization devices (multisite pacemakers) implanted, from June 2009 to March 2017, and who were admitted to Santa Helena's Hospital were analyzed.

This study was conducted according to the standards of the National Health Council (NHC) on research involving humans, as stated in the Resolution 446, adopted December 12^th^, 2012^[22]^p>. In this manner, the study was approved by the Ethics Committee of the Pontifícia Universidade Católica de Goiás (CAAE 46047915 2 0000 0037).

Data were obtained by reviewing the electronic medical records accessed via doctor's office - 2014 program and recorded in individual files. The study sample is not probabilistic, so the records were consecutively included.

The inclusion criteria were as follows: patients of both sexes with records available; patients diagnosed with CCC and over 18 years old; patients with NYHA class II, III or IV; and patients who have been using CRT for at least six months. Patients who did not meet the inclusion criteria were excluded as well as patients with incomplete medical records.

The study variables were as follows: age; sex; CRT duration; comorbidities (hypertension, diabetes mellitus, smoking, other comorbidities, and the use of an implantable cardioverter defibrillator [ICD]); medications (including beta-blockers, angiotensin-converting enzyme [ACE] inhibitor, angiotensinreceptor blockers [ARB], loop diuretics, thiazide diuretics, spironolactone, amiodarone, and digoxin); mortality; cause of death; number of hospitalizations; NYHA functional class before and after CRT; LVEF by Simpson before and after CRT; QRS duration before and after CRT; and arrhythmias and blocks (left bundle branch block [LBBB], right bundle branch block [RBBB], and left anterior fascicular block[LAFB]).

According to Brazilian guidelines^[6,14]^p>, patients with RBBB and RBBB+LAFB must undergo cardiac magnetic resonance or tissue Doppler echocardiography to establish the interventricular and intraventricular dyssynchrony before receiving the CRT system implant.

To classify patients as responders after six months using CRT, the criteria were as follows: 1) reduction of at least one point in NYHA class and 2) reduction of 15% in left ventricular end systolic volume (LVESV).

### Statistical Analysis

The data were collected and entered into a database using SPSS software, version 18 (SPSS Inc. Released 2009. PASW Statistics for Windows, Version 18.0. Chicago: SPSS Inc). Descriptive statistics were used to characterize the study sample.

Continuous variables were expressed as the mean ± standard deviation. Qualitative variables were evaluated using Chi-square test. Pearson's correlation was also used to measure the degree of linear relationship between two quantitative variables.

Appropriate tests were used to compare the variables as means (Student's t-test). Survival curves were generated using the Kaplan-Meier method. The results were presented as the hazard ratio (HR) with 95% confidence interval (95% CI). A *P* value of <0.05 was considered significant in all tests.

## RESULTS

We analyzed the medical records of 50 patients with CCC undergoing CRT, with an average CRT duration of 61.2±21.7 months. Of the 50 patients evaluated, 56% were male and 44% were female. The mean age of the patients was 63.4±13.3 years (Table 1).

**Table 1:** Patients' characteristics.

Characteristics		No. (%)	Mean	Standard Deviation
Sex	Female	22 (44)		
	Male	28 (56)		
Age (years)			63.4	13.3
Personal history	Hypertension	21 (42)		
	Diabetes mellitus	9 (18)		
	Current cigarette smoking	10 (20)		
	Chronic kidney disease	6 (12)		
	Coronary artery disease	5 (10)		
	Hypothyroidism	4 (8)		
	Previous stroke	2 (4)		
	Hypercholesterolemia	1 (2)		
	Megacolon	1 (2)		
	Megaesophagus	1 (2)		
	Chronic obstructive pulmonary disease	1 (2)		
	Polyneuropathy	1 (2)		
	Epilepsy	1 (2)		
Medication	Beta-blockers	32 (64)		
	ACE inhibitor	18 (36)		
	ARB	24 (48)		
	Spironolactone	22 (44)		
	Loop diuretics/thiazide	34 (68)		
	Amiodarone	35 (70)		
	Digoxin	15 (30)		
	Others	29 (58)		
Mortality	Cardiovascular cause	7 (14)		
	Other causes	6 (12)		
CRT use duration (months)			61.2	21.7
ICD use		37 (74)		

ACE=angiotensin-converting enzyme; ARB=angiotensin-receptor blockers; CRT=cardiac resynchronization therapy; ICD=implantable cardioverter defibrillator

Regarding comorbidities, 42% of the patients presented with hypertension, 18% had diabetes mellitus, 20% were current cigarette smokers and 46% had other comorbidities (Table 1).

Regarding medications, 64% of patients used beta-blockers, 36% used ACE inhibitors, 48% used ARB, 68% used loop diuretics or thiazide diuretics, 44% used spironolactone, 70% used amiodarone, 30% used digoxin, and 58% used some other drug (Table 1).

The use of ICD was reported in 74% of patients (Table 1). These patients had indications for the use of ICD before CRT, such as the presence of complex ventricular arrhythmias, results of electrophysiological study and secondary prevention.

The electrophysiological disturbances, like the presence of arrhythmias and bundle branch blocks, are shown in Table 2. Arrhythmias were identified in patients; nonsustained ventricular tachycardia (VT) was the most frequently observed arrhythmia (22% of patients), followed by paroxystic atrial fibrillation/ paroxysmal supraventricular tachycardia (PSVT) (14%) and sustained VT (6%).

**Table 2:** Electrophysiological disturbances of the patients.

Type		Frequency No. (%)	Frequency of responders No. (%)	Frequency of nonresponders No. (%)
Arrhythmia	Ventricular arrhythmia	2 (4)	2 (4)	__
	Paroxistic atrial fibrillation/PSVT	7 (14)	5 (10)	2 (4)
	Atrial flutter	1 (2)	__	1 (2)
	Sustained VT	3 (6)	3 (6)	__
	Nonsustained VT	11 (22)	7 (14)	4 (8)
	Not reported	26 (52)	22 (44)	4 (8)
Blocks	RBBB	5 (10)	3 (60)	2 (40)
	LBBB	15 (30)	12 (80)	3 (20)
	LBBB (dual chamber pacing)	15 (30)	10 (66.6)	5 (33.3)
	RBBB + LAFB	15 (30)	8 (53.3)	7 (46.6)

LAFB=left anterior fascicular block; LBBB=left bundle branch block; PSVT=paroxysmal supraventricular tachycardia; RBBB=right bundle branch block; VT=ventricular tachycardia

Sixty percent of the patients had LBBB, while 10% had RBBB; 30% of the patients had RBBB associated with LAFB. The expressive number of LBBB is due to dual chamber pacing patients which had done an upgrade to biventricular pacing (30%). Only 8 out of 30 patients with LBBB (33,3%) did not exhibit a CRT response. Additionally, 2 out of 5 patients with RBBB (40%) did not also exhibit a CRT response and 7 out of the 15 patients with RBBB + LAFB (46,6%) were also considered nonresponders.

The average QRS durations were 150.12±12.4 ms and 116.04±2.24 ms before and after CRT, respectively, showing a statistically significant difference (*P*<0.001). Only 1 (2%) patient presented a narrow QRS complex (QRS = 139 ms) before and after therapy. A total of 26 (52%) patients who had presented with a wide QRS complex (QRS >150 ms) before CRT exhibited a change to a narrow QRS complex (QRS <120 ms) after CRT. However, despite this change, 5 of these patients (10%) were considered nonresponders.

**Fig. 1:**
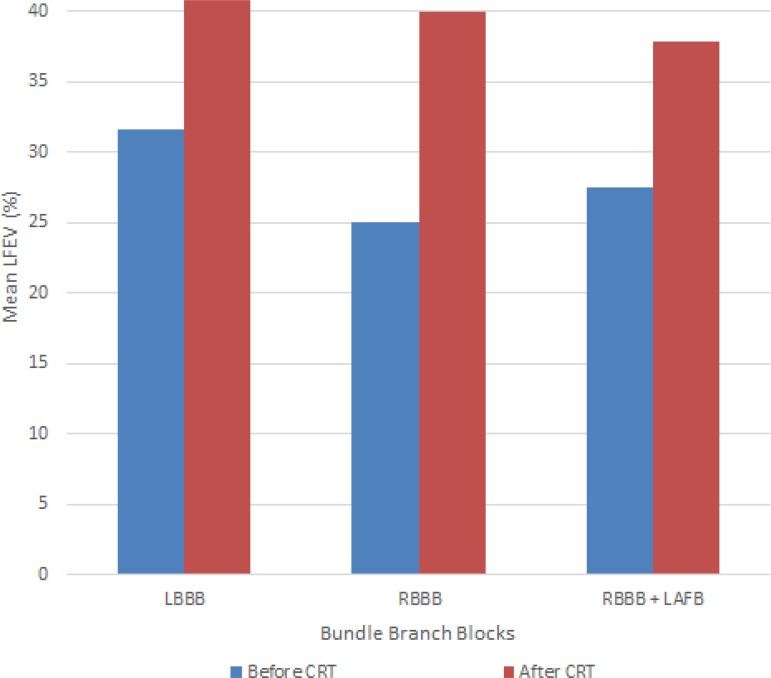
Mean left ventricular ejection fraction (LVEF) according to the bundle branch blocks presented by the patients before and after cardiac resynchronization therapy (CRT). LAFB=left anterior fascicular block; LBBB=left bundle branch block; RBBB=right bundle branch block

The mean LVEF before CRT was 29±7% and the mean LVEF after CRT was 39.9±13.1%, indicating a statistically significant increase in LVEF after CRT (*P*<0.001) (Table 3). Differently, the LVESV before CRT was 265±59 ml and the mean LVESV after CRT was 152±59 ml (*P*<0.01). The mean LVEF of the patients stratified by electrogram abnormalities (LBBB, RBBB, and RBBB+LAFB) before and after CRT are shown in Figure 1.

**Table 3:** Left ventricular ejection fraction before and after CRT use.

LVEF	Mean	Standard Deviation	*P* Value *
Before CRT	29%	7	
After CRT	39.9 %	13.1	<0.001

CRT=cardiac resynchronization therapy; LVEF=left ventricular ejection fraction

* *P* value estimated by Student's t-test.

**Fig. 2:**
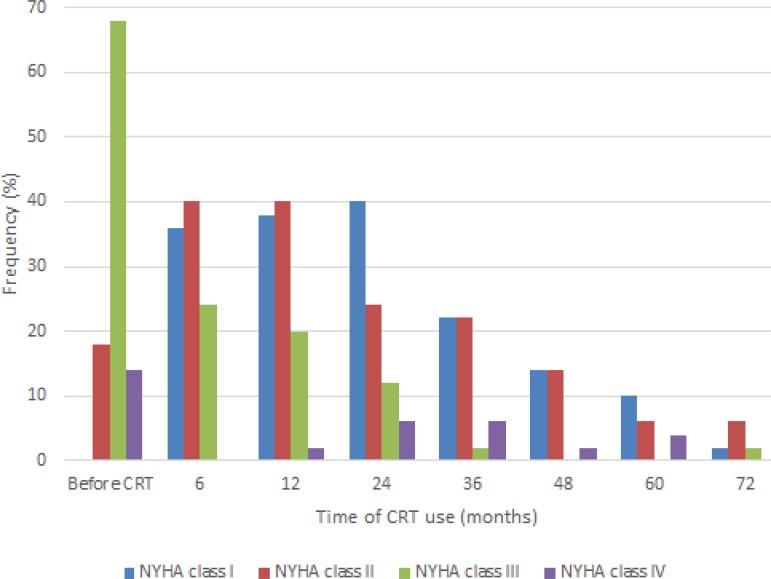
Variation of New York Heart Association (NYHA) functional class presented by the patients over the cardiac resynchronization therapy (CRT) duration.

With the implementation of CRT, there was a significant improvement in the NYHA functional class. As shown in Figure 2, the proportion of patients in NYHA classes III and IV decreased after 24 months of CRT (from 68 to 12% and from 14 to 6%, respectively) (*P*=0.014). Due to variations in CRT use duration, the data from 24 months onwards does not correspond to the total number of patients in the study, although there is a significant improvement in NYHA class variation over this time.

Based on evaluation of the improvement in functional class and LVESV individually, 66.6% of patients showed improvement in functional class after six months of CRT. However, 33.3 % did not exhibit any change in functional class and LVESV after six months of CRT.

During the use of CRT, 46% of responder patients required hospitalization, with an average of 1.5 (±2.2) hospitalizations. The following were the main causes of hospitalization presented by the patients in the study: ventricular arrhythmias, atrial arrhythmias, HF exacerbation, appropriate and inappropriate ICD therapies, pneumonia, chronic obstructive pulmonary disease, urinary tract infection, and decompensation of diabetes mellitus.

Death occurred in 25 responder patients (45%), 18 due to cardiovascular causes and 7 due to other causes (Table 1). Among the cardiovascular causes of death were arrhythmias (6%), stroke (2%), acute coronary syndrome (2%), and decompensation of congestive HF (acute pulmonary edema) (4%).

**Fig. 3:**
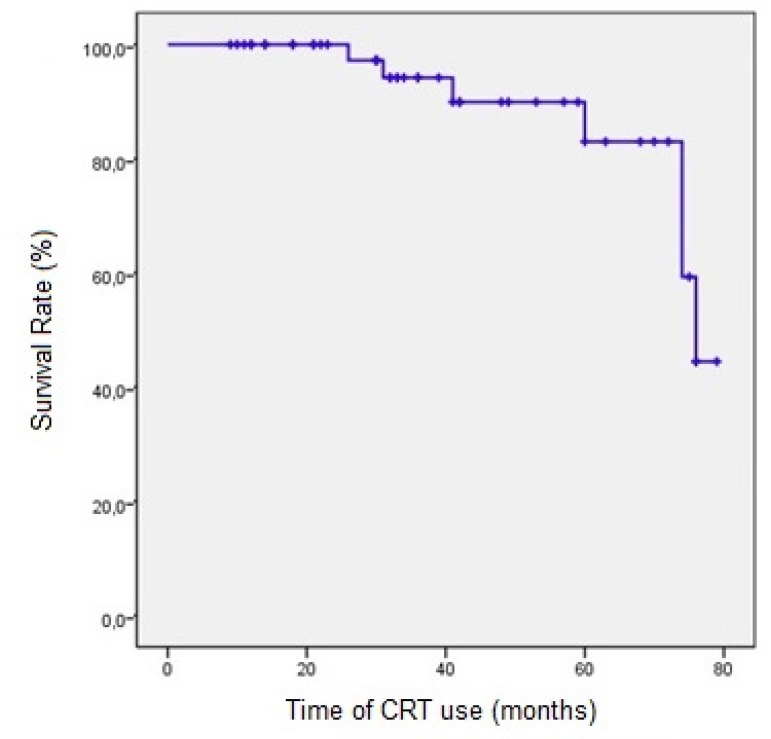
Kaplan-Meier estimates of survival for cardiac resynchronization therapy (CRT) responder patients.

The overall survival rate of responder patients estimated by the Kaplan-Meier method was 100% at 12 months and approximately 45% at 72 months. In the analysis adjusted by age (<40 years and ≥40 years), the survival rate was the same in both groups; therefore, there was no difference in response to the therapy due to age. However, no individuals under 40 years old had used CRT for 60 months (Figure 3).

## DISCUSSION

In our study, 56% of the patients were male, with mean age of 63.4 years, as observed in the literature. One study presented a higher percentage of males, with mean age ranging from 57.6 to 67.8 years. In another study, the mean age (53±12.1 years) was significantly lower in patients with Chagas disease than in those with other etiologies (*P*<0.001)^[23-25]^p>.

In the literature, the main heart conditions associated with CCC are complex ventricular arrhythmias; co-occurring tachyarrhythmias and bradyarrhythmias; and biventricular HF^[23]^p>. There is also a combination of LBBB and LAFB that is quite typical of CCC^[3]^p>. In our study, 48% of patients showed some arrhythmia, with 22% exhibiting nonsustained VT and 6% exhibiting sustained VT. Regarding blocks, 20% had RBBB, 46% had LBBB, and 32% had RBBB+LAFB.

Studies estimate that a QRS duration of ≥120 ms occurs in 25-50% of patients with HF^[26,27]^p>. In the present study, 98% of patients already had a QRS duration of >150 ms before the use of CRT, with an average QRS duration of 150.12 ms. After the use of CRT, that average dropped to 116.04 ms, a significant difference (*P*<0.001) following large studies, such as COMPANION and MADIT-CRT, and showing that patients with a long QRS duration benefit from CRT^[7,12]^p>.

Our study showed that 33.3% of patients did not respond to CRT. A study of 72 patients with CCC showed that 65.3% of them responded to CRT at the end of a follow-up ranging from 4 to 79 months^[25]^p>. Another study evaluating the use of CRT in patients with CCC reported that 95.3% of them (20 patients) were responders and that only 4.7% of patients (1 patient) did not respond to the therapy^[28,29]^p>.

Several studies consider NYHA functional class as an evaluation parameter of the response to CRT^[30]^p>. In our study, variations of NYHA class over time showed that before CRT, 68% of patients were in NYHA class III, 18% in NYHA class II and 14% in NYHA class IV. After six months of CRT use, only 22% of patients (11 patients) showed no improvement in functional class. A study of the use of CRT in 21 patients with CCC, with a follow-up of over 18 months, showed that 85.7% of patients had improvements in functional class^[31]^p>. In the study by Martinelli Filho et al.^[25]^p>, 83.8% of patients showed improvement by at least one functional class, while 16.2% remained in the same functional class or worsened it.

In the study of Araújo et al.^[31]^p>, it was possible to verify clinical benefits of the use of CRT in patients with CCC and at the end of their follow-up, they showed statistically significant improvements in functional class and LVEF as well as reduced left ventricular diameter and end-systolic volume, in agreement with our results.

Our study showed a significant increase in the mean LVEF before and after the use of CRT. In agreement with our finding, other studies also showed a significant increase in this parameter^[28,31]^p>.

A systematic review of predictors of mortality in CCC revealed that symptoms of HF (NYHA class III/IV), left ventricular impairment on echocardiography, cardiomegaly on chest radiography and nonsustained VT events in Holter are factors associated with the risk of death or a worse prognosis; therefore, these symptoms require stricter interventions^[23]^p>.

In our study, the survival rate after 72 months of CRT was 45%, which was lower than the rate of 50% estimated in the literature after 60 months from the diagnosis of HF^[14,32,33]^p>.

Several studies have shown that up to two-thirds of patients with HF are hospitalized on average twice a year, and the rate of rehospitalization within 3 months reaches 20-30%^[34]^p>. In our study, the number of hospitalizations was not related to the CRT use duration (*P*=0.548), but it was significantly inversely associated with cardiovascular cause of death (*P*<0.001).

Another study of the use of CRT with or without ICD showed significant benefits of the isolated use of CRT and ICD in HF. However, the effect of the combination of these therapies is not well established as the study data are still insufficient^[35]^p>.

Rassi et al.^[23]^p> state that one of the main causes of death in Chagas disease is HF. In a study of CCC patients using CRT with long-term follow-up, the overall mortality was 34.7% (25 patients). Of these deaths, 76% were due to cardiovascular causes, 8.3% due to non-cardiovascular causes and 16% due to unknown causes. The worsening of HF was the main cause of death among the cardiovascular causes (60% of the cases), in agreement with our results^[31]^p>.

Martinelli Filho et al.^[25]^p>showed that, at the end of the proposed follow-up, 97 (28.2%) deaths were recorded, corresponding to an annual mortality of 6.7%. The cardiac cause was the main cause of death, corresponding to 84.5% of the known causes. The authors noted that this increased mortality may have been related to the high number of patients with advanced atrioventricular block, and they state that although CRT has a positive impact on the clinical behavior of patients with CCC, the long-term prognosis is worse compared to other heart diseases^[25]^p>.

### Limitations

Our study has some limitations. First, it was a single-center study that included a limited number of patients. Furthermore, being a retrospective study, the data were derived from medical records, however, all patients in accordance with inclusion criteria were studied.

## CONCLUSION

This study revealed that a relevant number of patients with CCC using CRT has been considered as CRT responders (reduction of the NYHA functional class and reduction of LVESV), otherwise, these patients had no significant improvements in survival rates after 72 months.

**Table:** 

Authors' roles & responsibilities	
ASMJ	Designed the study and performed the experiments and wrote the manuscript; final approval of the version to be published
CCL	Designed the study and performed the experiments and wrote the manuscript; final approval of the version to be published
PFC	Performed the experiments; final approval of the version to be published
EM	Analyzed the data; final approval of the version to be published
